# Cost comparison of two approaches to chiropractic care for patients with acute and sub-acute low Back pain care episodes: a cohort study

**DOI:** 10.1186/s12998-020-00356-z

**Published:** 2020-12-14

**Authors:** James M. Whedon, Serena Bezdjian, Patricia Dennis, Vivi-Ann Fischer, Robb Russell

**Affiliations:** 1 Southern California University of Health Sciences, 16200 Amber Valley Drive, Whittier, CA 90604 USA; 2 Fulcrum Health, Inc, Plymouth, MN USA

**Keywords:** Acute or subacute low back pain, Chiropractic care, Cost comparison, Patient care

## Abstract

**Background:**

Low back pain (LBP) imposes a costly burden upon patients, healthcare insurers, and society overall. Spinal manipulation as practiced by chiropractors has been found be cost-effective for treatment of LBP, but there is wide variation among chiropractors in their approach to clinical care, and the most cost-effective approach to chiropractic care is uncertain. To date, little has been published regarding the cost effectiveness of different approaches to chiropractic care. Thus, the current study presents a cost comparison between chiropractic approaches for patients with acute or subacute care episodes for low back pain.

**Methods:**

We employed a retrospective cohort design to examine costs of chiropractic care among patients diagnosed with acute or subacute low back pain. The study time period ranged between 07/01/2016 and 12/22/2017. We compared cost outcomes for patients of two cohorts of chiropractors within health care system: Cohort 1) a general network of providers, and Cohort 2) a network providing conservative evidence-based care for rapid resolution of pain. We used generalized linear regression modeling to estimate the comparative influence of demographic and clinical factors on expenditures.

**Results:**

A total of 25,621 unique patients were included in the analyses. The average cost per patient for Cohort 2 (mean allowed amount $252) was lower compared to Cohort 1 (mean allowed amount $326; 0.77, 95% CI 0.75–0.79, *p* < .001). Patient and clinician related factors such as health plan, provider region, and sex also significantly influenced costs.

**Conclusions:**

This study comprehensively analyzed cost data associated with the chiropractic care of adults with acute or sub-acute low back pain cared by two cohorts of chiropractic physicians. In general, providers in Cohort 2 were found to be significantly associated with lower costs for patient care as compared to Cohort 1. Utilization of a clinical model characterized by a patient-centered clinic approach and standardized, best-practice clinical protocols may offer lower cost when compared to non-standardized clinical approaches to chiropractic care.

**Supplementary Information:**

The online version contains supplementary material available at 10.1186/s12998-020-00356-z.

## Introduction

In the United States (US), low back pain (LBP) imposes a costly burden upon patients, healthcare insurers, and society overall. A systematic review published in 2008 estimated that expenditures for clinical care of patients with LBP ranged from $12 to $91 billion [1]. More recently, it was reported that spending for low back and neck pain had become accountable for the highest costs in US health care, with an estimated spending of $134.5 billion [2]. The total economic burden for LBP (not limited to expenditures alone, but also including indirect costs, such as disability and loss of productivity) is uncertain, but could range as high as $625 billion [1]. The escalating clinical costs of spine care have been compounded by adverse consequences of the epidemic of opioid prescribing in the US [3]. Among US adults prescribed opioids, 59% reported having back pain [4]. Current evidence-based clinical guidelines for first-line treatment of low back pain now recommend avoidance of opioid prescribing in favor of non-pharmacological therapies, including spinal manipulation, which is widely provided by chiropractors [5].

From 1999 to 2008 mean inflation-adjusted annual expenditures on medical care for patients with back and neck conditions increased by 95%, while expenditures on chiropractic care remained relatively stable [6]. Stable overall costs suggest that chiropractic care may offer savings [7], but differences in cost per patient and per episode remain uncertain. A study of Medicare claims data found that among more than 72,000 older multimorbidity patients with chronic LBP, those who received chiropractic care had lower overall costs of care - and lower cost of care per episode day - than patients who received conventional medical care [8]. A 2015 systematic review reported overall lower costs for chiropractic care, but costs were higher in studies that also examined clinical outcomes [9]. A 2016 systematic review was also inconclusive regarding whether chiropractic or medical care is more cost-effective for the treatment of low back pain [10]. A more recent systematic review reported that spinal manipulation therapy is likely to be cost-effective for treatment of LBP [11]. However, there is wide variation among chiropractors in their approach to clinical care, and it is not certain what is the most cost-effective approach to chiropractic care of low back pain. Little has been published on the comparative costs of different clinical practice approaches to chiropractic care. Thus, the present study was designed to compare costs of clinical care for acute or sub-acute care episodes for low back pain across two groups of providers organized within a network of chiropractor providers.

### Chiropractic provider network

We studied patient encounter data provided by a nonprofit physical medicine management organization that was founded in 1984 and is based in the Midwestern US. The healthcare system provides care for patients with spine related disorders through *ChiroCare*, a network of chiropractor providers and practicing locations. In 2015, the healthcare system created the *ChiroCare Centers of Excellence (CoE) Program* to recognize clinics with a validated use of standardized clinical protocols and utilize an integrated, collaborative approach for achieving positive outcomes and improved quality of life for patients. The discipline framework for the criteria of the CoE program was developed by a task force on spine care with various specialties and stakeholders – primary care physicians, orthopedic surgeons, physical therapists, acupuncturists, Doctors of Chiropractic, and industry leaders. The criteria, or Attributes of Excellence for CoE clinics include comprehensive patient intake and history, use of assessment and outcome measurement tools, addressing biopsychosocial issues, construction of patient-centered treatment plans with measurable functional goals, patient engagement with shared decision making, conservative use of imaging, active care, self-care and preventative instructions, care coordination between practitioners, and patient education and empowerment for self-efficacy. The present study was designed to compare costs between Centers of Excellence and other ChiroCare providers. This cost study was one component of a larger research project investigating chiropractic care for LBP regarding its impact on the Triple Aim (cost, outcomes, and patient satisfaction). The project involved development of a conservative care outcome measure, and measurement of patient satisfaction scores using the Clinician and Group Consumer Assessment of Healthcare Providers and Systems (CG CAHPS). Within the scope of the larger project, the objective of the present study was to compare expenditures for the treatment of acute or sub-acute care episodes for LBP across the two tiers of the chiropractic provider network, to determine if a patient-centric, center of excellence model resulted in lower costs. The next section briefly outlines the two tiers of providers with the healthcare system.

### Two tiers of providers

The ChiroCare Network (CC) of providers is the largest of two tiers of providers and is comprised of approximately 2300 doctors (i.e., Doctor of Chiropractic). Centers of Excellence (CoE) is the second tier and is comprised of approximately 117 doctors. CoE providers only achieve this designation upon completion of a rigorous initial external review and annual verification, of clinical practices and protocols to validate alignment with the following distinguishing characteristics: (1) they lead with evidence-based quality care; (2) use a comprehensive history and intake process; (3) apply shared decision making with the patient to identify measurable treatment goals; (4) address identified biopsychosocial factors; (5) comprehensive exam; (6) conservative use of plain film imaging; (7) active care exercises; (8) clinic process established for referrals and coordination of care; (9) home instructions; (10) patient education to empower self-efficacy; and (11) wellness and prevention instructions (see Additional file 1: Appendix for a full description of the distinguishing attributes of the Centers of Excellence).

As a result of these distinguishing characteristics and patient-centered approach, we hypothesized that CoE Providers would be significantly associated with lower clinical costs compared to the larger CC Network of providers.

## Methods

We employed a retrospective cohort design to analyze health insurance claims data provided by the health network. The datasets consisted of paid claims containing information on providers, health plan members, and clinical encounters. The data were analyzed primarily for descriptive statistics, trends in expenditures, and exposure factors affecting expenditures. The study was conducted in accordance with all ethical research standards and approved by the Institutional Review Board, Southern California University of Health Sciences.

### Population

The study population included adult patients aged 18 years of age or older with claims for chiropractic care delivered through the ChiroCare Network, with health plan coverage for chiropractic services. We included health plan members with a new primary diagnosis of LBP (as defined by any one of a selected constellation of diagnosis codes) and an acute or sub-acute episode duration. The rationale for restricting the study to acute and sub-acute care was to differentiate newly diagnosed patients from those with chronic pain, for whom the approach to care may differ.

### Inclusion and exclusion criteria

The time period covered by the cost analysis ranged between 07/01/2016 and 12/22/2017. New patients 18 years of age or older with at least two claims for a primary diagnosis of LBP within 90 days and a treatment start date occurring 07/01/2016 and later were included in the analyses. We utilized a look-back period of 180 days to exclude patients with a history of low back pain prior to 07/01/2016. Patients with comorbid diagnosis of cancer, trauma, drug abuse, or infections of the spine were also excluded. Patients with chronic low back pain (i.e., with episode duration greater than 90 days) were also excluded. Prior to analysis, the dataset was subjected to extensive data cleaning operations intended to eliminate erroneous data (e.g., duplicate claims) and enhance validity, application of exclusion criteria, assembly of cohorts, and definition of episodes of care.

### Cohort assembly

All eligible providers held a Doctor of Chiropractic (DC) degree. The providers were categorized according to their tier within the ChiroCare Network. By association with providers seen at clinical encounters, patient subjects were assembled into two mutually exclusive cohorts: CC Network (Cohort 1), and CoE (Cohort 2). There were no differences between the two cohorts in the types of services covered or in re-imbursement rates of private and public insurance plans. No patient crossover between provider categories was observed in the data.

### Statistical analysis

All statistical analyses were computed using IBM Statistics Software SPSS (Version 23) [12] and all data management procedures followed IRB guidelines. Claims data were examined using the procedure codes as defined by Current Procedural Terminology (CPT) Code. Procedures were categorized as evaluation and management, manipulative therapy, physiological therapeutics, acupuncture, and imaging. The physiological therapeutics category was divided into active (e.g. therapeutic exercise, therapeutic activities, and neuromuscular re-education) and passive (e.g. electric muscle stimulation, ultrasound, massage therapy, manual therapy, and traction). Claims for manipulative therapy of extremities (e.g., 98,943; pertaining to the wrist or knee) were removed from the analyses because such procedures are typically not directly related to treatment of low back pain. Thus, only chiropractic treatments for low back pain were included in the analysis; treatments provided by other types of practitioners were not included. Additionally, because all providers in the current study were chiropractors, CPT 97140 was likely used for manual therapies other than manipulation.

We generated descriptive statistics including means and frequencies on patient age, gender, and other clinical factors (health plan, health plan benefit packages, provider region, and primary diagnosis categories). We categorized health plan benefit packages as either public (benefit packages structured for Medicare or Medicaid populations where costs are at least in part funded by State or Federal government entities) or private (benefits structured for Commercial group or individual populations where costs are privately funded by an employer or an individual). Provider Region was dichotomized as Urban versus Rural). Urban regions are comprised of larger metropolitan areas, while rural regions are comprised of smaller towns or cities. Primary diagnosis of low back pain was categorized as *biomechanical lesions*, *sprains*, *strains*, *or other dorsopathies*.

Generalized linear modeling (GLM) (with gamma distribution and a log link function) was used to estimate the influence of patient and clinician characteristics on costs, as measured by allowed amounts. Studies examining cost data have demonstrated that the GLM model with the gamma distribution is an optimal choice when modeling skewed cost data [13, 14]. Variables with a *p*-value *p* < .05 were considered statistically significant.

## Results

### Descriptive statistics

Table 1 presents patient characteristics for each of the two cohorts examined. In the current study’s sample, there were a total of 1436 providers in the CC Network and 81 CoE providers. The 25,621 unique patients included in the analyses were associated with 220,079 clinical encounters. Subject age ranged from 18 to over 90 years of age with a mean age of approximately 50 years old (for males and females) in the two cohorts. Table 1 also presents distributions for Health Plan, Provider Region (Urban and Rural), and Primary Diagnosis Category. Distributions for Health Plan (*χ*^2^ = 8.34, *p* = .004), Region (*χ*^2^ = 533.9, *p <* .001), and Primary diagnoses (*χ*^2^ = 139.67, *p* < .001) were statistically different between the two cohorts. More than 22,000 unique patients were seen by CC providers, the largest of the two mutually exclusive provider cohorts.

**Table 1:** Sample Characteristics *[N* = 25,621 unique patients*]*

Cohort of Providers	Mean Age in Years by Gender	Gender (% Female)	Health Plan‡	Region (Urban vs. Rural)†	Primary Dx*
ChiroCare Network (CC) (22,066 patients)	47 (females) 48 (males)	55% (*n* = 12,079)	70% Private 30% Public	65% (Urban) 32% (Rural) 3% (Missing)	85% Biomechanical Lesions 9% Other Dorsopathies 3% Sprains 3% Strains
Centers of Excellence (3555 patients)	48 (females 49 (males)	59% (*n* = 2079)	68% Private 32%Public	86% (Urban) 14% (Rural)	78% Biomechanical Lesions 14% Other Dorsopathies 6% Sprains 2% Strains

Note. the ‘Over 90’ age category is not reflected in patient mean age; ‡ Significant difference in Health Plan between the two Cohorts χ2 = 8.34, p = .004; †Significant difference between the two Cohorts on Region; χ2 = 533.9, p < .001; *Significant difference between the two Cohorts on Primary Dx (based on first visit) χ2 = 139.67, p < .001

**Fig. 1:**
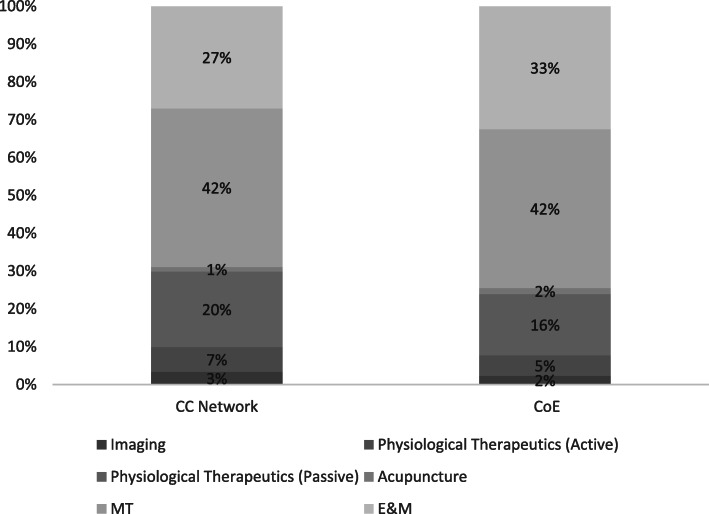
Distribution of Current Procedural Terminology (CPT) Codes by Cohort per Episode. MT – manipulative therapy; E&M – evaluation and management; CC Network – ChiroCare Network; CoE – Centers of Excellence

Figure 1 displays the distribution of key CPT codes for the two Cohorts (CC and CoE). As exhibited in the Figure, there are slight differences in the distribution of the CPT codes between the two cohorts. Most notably, we see slightly more procedures associated with Acupuncture and Evaluation and Management (E&M) and slightly less associated with imaging and physiological therapeutics (both active and passive) in the CoE cohort. [Fig. 1] The higher utilization rate observed associated with E&M codes is part of a patient-centered approach, where the CoE chiropractor is focused on the patient’s progress to meet measurable treatment goals and re-evaluates to modify care as indicated to further achieve those goals.

Table 2 displays the results of regression modelling for Allowed Amounts. Compared to CC providers, CoE providers were associated with statistically significantly lower costs for *allowed amounts* (.77, 95% CI 0.75–0.79, *p* < .001), which translates to approximately 23% lower costs ($252 vs. $326). Additionally, when compared to males, females were associated with slightly higher allowed amounts [1.04, 95% CI 1.02–1.05, *p* < .001]. Health Plan and Provider Region also significantly influenced cost (allowed amounts): private plans were approximately 50% higher in cost than public plans ($350 vs. $235) and urban areas were 23% higher in cost compared to rural areas. Table 2 also displays the mean allowed dollar amounts along with the 95% CIs for each of the factors in the model. [*Note*. we also tested a model with age and primary diagnoses, but those factors did not significantly influence cost and thus were dropped from the model. Thus, we present a simplified version in Table 2.]

**Table 2:** Effect of Clinical and Demographic Factors on Allowed Amount Per Patient Per Episode

Factor/Exposure	Group/Referent	(exp) Allowed Amount	95% CI	p-value	Mean Amounts Group/Referent	95% CI (Mean Amounts) Group/Referent)
**Intercept**	–	237	231.8–242.3	*p* < .001	–	
**Cohort**	CoE/Network	0.77	0.75–0.79	*p* < .001	$252/$326	($246 - $258)/($323 - $330)
**Health Plan**	Commercial/Gov’t	1.49	1.47–1.52	*p* < .001	$350/ $235	($345 - $355)/ ($230 - $239)
**Region**	Urban/Rural	1.23	1.21–1.25	*p* < .001	$318/$259	($313–$322)/($254 - $264)
**Sex**	Female/Male	1.04	1.02–1.05	*p* < .001	$292/$282	($287–$296)/($277–$286)

*Note. Dependent Variable = Allowed Amount (positive expenditures modeled; N = 24,330). Exposure variables in this model include: Cohort, Health Plan, Region, and Sex (entered as factors in the GLM). maximum likelihood estimate*

*Exposure of interest/reference group (variable). Reference (or comparison) group is specified after the forward slash “/”*

*Mean (allowed) amounts presented for Group/Referent and 95% CIs (for mean allowed amounts) come from the GLM*

*model’s estimated mean*

## Discussion

The findings of this study support our hypothesis that CoE chiropractors provide care at a significantly and appreciably lower clinical cost, as compared to CC providers. Although we cannot say definitively, the lower expenditures may be attributable to the characteristics of the CoE providers. Our findings suggest that implementation of a patient-centered approach with standardized, best-practice clinical protocols may lead to cost savings in the care of patients with acute or sub-acute low back pain. For this unique population of patients and providers, there is no valid means of comparing the results to other chiropractic care networks or conventional medical care networks. However, our findings do appear to provide additional weight to the evidence in favor of adopting a comprehensive, conservative care pathway to reduce costs for the primary care of patients with spinal pain disorders even within chiropractic care settings. This general approach has been described as the Primary Spine Care model of care for patients with spine related disorders [15]. Primary Spine Practitioner is a first-contact provider for patients with spinal problems, for practitioners that desire to work in a team-based environment that comprehensively manages and coordinates the care for individuals with these spine-related disorders. The practice of Primary Spine Care has been reported to decrease variability in care, decrease costs, and improve outcomes [16–18].

Despite increased intervention and enormous costs, there has not been an appreciable decrease in the incidence or prevalence of LBP. Similarly, clinical outcomes and disability associated with LBP have not improved and, in the case of the latter, have worsened. The lack of widely accepted and clearly articulated approaches to successful management of LBP is a source of confusion for healthcare practitioners, payers, and patients alike. With the understanding that LBP is often not successfully managed under the prevailing model of care, the implementation of best-practice approach, as exemplified by the Centers of Excellence, may bring meaningful improvements to clinical outcomes, delivery, and affordability. When a chiropractor is a first point of contact within the delivery system, chiropractors diagnose the patient’s condition and triage the patient to the right care. Many patients find benefit and relief from LBP with effective treatment from a chiropractor and save unnecessary expense and time to navigate a complexity of treatment options and provider specialty types. The CoE chiropractor demonstrated focus on evidence-based treatment options and patient-centered care, with attention to the needs of the individual patient and the treatment options that will best help. Within CoE practices, there is demonstrated alignment with primary care providers and other members of the patient’s care team, ensuring a team-based approach. CoE providers in Cohort 2 are process oriented providing consistency of care and quality standards for each patient.

This study demonstrates the potential positive economic impact of chiropractic care using a best-practice approach. While this study was limited to the care of patients with acute or subacute episodes of LBP, the care model described may be applicable to care pathways for chronic spine pain disorders such as chronic LBP.

### Limitations

Our study has several limitations. First, the findings from this healthcare system were limited to examining patients with an episode of acute or sub-acute low back pain and may not be generalizable to other healthcare systems or to the U.S. population. Additionally, cost comparisons among patients with chronic low back pain may be different from patients with an acute or sub-acute episode. Because this study was a non-randomized observational study, it is not possible to draw causal inferences regarding the relationship between exposures and outcomes; unmeasured or unknown confounders may be responsible for the observed associations. Additionally, we did not have claims data from non-chiropractic providers; thus, the scope of this study was limited to cost comparisons between two approaches to chiropractic care. In the present study, exposure factors in the GLM model included the cohort designation, health plan, provider region, and patient sex; future research should model and examine clinical outcomes, or baseline status, which may have influenced cost outcomes. Cognitive behavioral therapy (CBT), one of the attributes of the approach to care listed of CoE providers, (Additional file 1: Appendix) was not a covered service; thus, costs for CBT were not included in the analysis. The goal of this analysis was to identify practice patterns that result in differences in allowed costs, which are a valid measure of costs borne by the patient and the third-party payor combined. A truly comprehensive assessment of costs is beyond the scope of this project because it would require additional assessments of expenses (e.g., patients’ personal expenses, business costs). Despite these limitations, the internally valid cost comparison resulting from this study provides a significant contribution to understanding the value of pursuing a rigorously evidence-based approach to chiropractic care. Building upon evidence that favors non-pharmacological care as the preferred approach to treatment of spinal pain, future research should continue to focus on identifying specific approaches to chiropractic care that offer the greatest patient benefit at the lowest cost.

## Conclusion

After adjusting for demographic factors, we found evidence of significantly lower costs per patient for chiropractors using a patient-centered clinic approach and standardized, best-practice clinical protocols. Despite modest effect sizes, such an approach could result in significant cost differences in the care of patients with low back pain. If this model of care provides equivalent or better outcomes, it may offer superior value as compared to general chiropractic care. Future research should focus on identifying the specific characteristics of evidence-based chiropractic practice that affect cost outcomes. Other healthcare systems may want to employ a similar model to examine the value of a patient-centered, conservative care pathway for management of patients with low back pain.

## Supplementary Information

**Additional file 1.** Attributes of the Centers of Excellence.

## Data Availability

The research datasets are proprietary and therefore not publicly available. Furthermore, release of the health claims data used for this study is disallowed due to the mandate for protection of patient privacy and confidentiality.
