# Supplementary material for: Cost comparison of two approaches to chiropractic care for patients with acute and sub-acute low Back pain care episodes: a cohort study
Source: Chiropr Man Therap. 2020 Dec 14;28:68. doi: 10.1186/s12998-020-00356-z (PMC7734754; doi:10.1186/s12998-020-00356-z)
Supplement: Supplementary file 1 — Additional file 1. Attributes of the Centers of Excellence. [file 12998_2020_356_MOESM1_ESM.docx]

**APPENDIX**

**Attributes of the Centers of Excellence**

| **Attribute** | **Description** |
| --- | --- |
| 1. Comprehensive Patient Intake and History | Comprehensive patient intake and history to identify the medical context of the patient’s condition, including the onset of care (initial, recurrent, continuation, acute, chronic) and prior care received. Co-morbidities are identified and considered in the development of the plan. |
| 1. Assessment and Outcome Tools | The Center of Excellence clinician uses standard assessment tools to document and track patient progress, which is critical in formulating an effective treatment plan and demonstrating the overall effectiveness of the care. Examples of tools available for measurement of a patient’s progress are standardized functional and pain measurement tools such as Numerical Rating Scale, Oswestry, Neck and Low Back Indexes; and psychosocial assessment tools such as PHQ-9 and the Keele STarT Back. Administration of assessment tools at the initial visit and throughout treatment provides insight regarding the patient’s status. |
| 1. Cognitive Behavioral Therapy | In considering the patient’s physical, social and psychological situation, there may be opportunities to provide patient-centered counseling for eliciting behavior change by helping clients to explore and resolve ambivalence. This coaching provides step by step encouragement to healthier lifestyle activities. |
| 1. Shared Decision Making | Centers of Excellence doctors demonstrate patient communication skills that include active listening and shared decision-making regarding goals and options for treatment. |
| 1. Conservative Imaging | Centers of Excellence adhere to the recommendations of the American Academy of Family Physicians and the *Choosing Wisely®* program by avoiding plain films and advanced radiology in the first six weeks of care in the absence of any red flags. After six weeks, lack of progress or worsening of condition could indicate reconsideration of imaging. |
| 1. Treatment Plans | A quality, best-practice Treatment Plan includes four key elements: Patient History Information (PHI), Diagnosis, Treatment Recommendations, and Re-evaluation. The treatment plan identifies the medical context of the patient’s condition, including the onset of care (initial, recurrent, continuation, acute, chronic) and the level of care. Based upon a review of the health history, screening and pain and/or functional measurement tools, the physical examination and consultation with the patient, the clinician establishes a diagnosis. The diagnosis must consider the patient’s physical, social and psychological situation. One of the goals of the diagnosis is to identify whether the patient can be treated by chiropractic care or if the patient should be referred to a specialist. After determining the diagnosis and consultation with the patient regarding their treatment options, a specific plan of treatment and recovery is developed. One of the primary benefits of a treatment plan is to establish goals and expectations for the patient regarding improvement and the level of care needed. Re-evaluation is an important part of the care planning process to assess functional progress or determine adjustment of the treatment based on changing conditions or symptoms. |
| 1. Coordination of Care | The care coordination process includes specific protocols designed to ensure that the patient successfully reaches the providers outside of the Center of Excellence’s office (i.e., when referring to a specialist, ordering tests or when instructed to follow-up with the patient’s primary care provider).  A valid Patient Consent Form includes, but is not limited to: consent to release information such as health records and medical information to physicians, providers and staff necessary for treatment, permission to communicate information about the patient to others involved in their care for the purpose of treatment, permission to communicate with the patient regarding medical care, and results of tests, etc. |
| 1. Care Management | Care management involves validating the patient’s engagement and compliance with their care plan. This includes processes to confirm that the patient is completing their active care exercises, following up when the patient misses an appointment and reviewing treatment plans and expectations with the patient throughout the care delivery.  Patients who fail to achieve measurable gains should be considered for a modified treatment plan, additional diagnostic evaluation and/or specialist referral, co-management or an alternative therapeutic approach. Appropriate pain management of spine-related conditions includes addressing the issues of physician dependence, somatization, illness behavior and secondary gain. |
| 1. Established referral list & process | Centers of Excellence support an integrated approach to patient care by establishing and maintaining referral network relationships. A Center of Excellence has strong working relationships with primary care practitioners, physical therapists, occupational therapists, behavioral therapists, orthopedic and neurological providers, surgeons and other practitioners. Documented referral procedures exist to ensure consistency and effectiveness of the referral process. The referral process includes patient education regarding the need for further evaluation and/or the benefits of recommended treatments. |
| 1. Active Care Instructions | A Center of Excellence provider is a proponent of the value of active care in the treatment plan and has materials and tools available for patients. Active care is the training of motor control patterns that protect the spine. The training is step by step instruction to improve stability and neuromuscular control, progressing to stabilization exercises, and finally to active conditioning. Key elements of active care include: reassuring the patient it is safe and beneficial to gradually resume activity, engaging the patient to share responsibility for their recovery, advice to reduce exposure to repetitive strain, educating on the benefits of long-term active care to reduce risk of chronicity, and assisting patients to achieve their goals for restored function. |
| 1. Home Care Instructions | Home Instruction should include steps the patient can do to facilitate their recovery such as heat, ice, durable medical equipment. These instructions may also include work instruction for preventing further injury, such as instructions on proper sitting, standing, bending, posture, and repetitive movements; as well as general engagement in self-management and future Injury prevention. |
| 1. Wellness Care Instructions | Materials and education are available regarding health information such as smoking cessation information, anti-inflammatory diet, weight loss, etc. to help the patient live a healthy lifestyle. |
| 1. Patient-centered Approach to care plans | A Center of Excellence clinician continually exhibits an understanding that the patient’s best interests are always of primary importance and utilize a comprehensive care plan approach unique to meeting the needs of the patient. The clinician actively communicates with the patient regarding their condition, treatment goals, treatment plan options, and progress using a Shared Decision-Making approach. |
| 1. Demonstrate collaboration with other practitioners | Centers of Excellence support an integrated approach to patient care by establishing and maintaining referral network relationships. A Center of Excellence has strong working relationships with primary care practitioners, physical therapists, occupational therapists, behavioral therapists, orthopedic and neurological providers, surgeons and other practitioners. Documented referral procedures exist to ensure consistency and effectiveness of the referral process. The referral process includes patient education regarding the need for further evaluation and/or the benefits of recommended treatments. |
|  |  |
|  |  |
